# Supplementary material for: Economic burden and cost-effectiveness of treatments for open tibia fractures in Malawi: Economic analysis of a multicentre prospective cohort study
Source: PLoS One. 2025 Sep 5;20(9):e0331569. doi: 10.1371/journal.pone.0331569 (PMC12413004; doi:10.1371/journal.pone.0331569)
Supplement: S1 File — (DOCX) [file pone.0331569.s001.docx]

**Economic analysis of open tibia fracture treatment in Malawi**

**Periodic Activity Review of facilities providing open tibia fracture care**

**Topic Guide for Information Collection**

**January 2021**

**Facility Name: ....................................................................**

**Contact/Focal Person: ....................................................................**

**Date of Activity Review: .....................................................................**

**Data Collection Period: .....................................................................**

1. **Introduction**

This periodic activity review is intended to document the nature, range and method of delivery of open tibia fracture treatment in each facility. Specifically, we would like to understand how service integration is working in this facility.

The periodic activity review has the following objectives:

1. To understand the organization and size of the facility.
2. To review activities and services currently being delivered in each facility.

3. To understand how integration of services provided in this facility works.

This instrument is designed for members of the economics team to collect information through interviews with key staff and observation of activities.

1. **Open tibia pathway for QECH initial hospital visit (confirm at each recruitment site)**

Injury

District hospital

Healthcare facility

First aid treatment

Exit/Discharge

TITAN recruitment

Minor theatre

(Debridement and immobilisation)

Xray

Ward 6A/5B (shared staff)

67%

(either HF or DH)

QECH Emergency department (shared staff)

Major theatre

(Debridement and internal fixation +/-soft tissue procedure)

1. **Breakdown of pathways and observations required by department (to be confirmed by staff interviews)**

**ATC emergency department pathway:**

1. Register patients to QECH
2. Assessing neurovascular status (not always done)
3. Cannulation
4. Administering antibiotics
5. Administering analgesia
6. Send patient to Radiology (see below)
7. POP

**Radiology department**

1. Check patient details
2. Position patient
3. Get anteroposterior view
4. Get lateral view

**Debridement**

1. Move to theatre table
2. Spinal anaesthetic
3. Clean “social scrub” limb
4. Prep limb
5. Drape patient
6. Excise non-viable tissue
7. Wash
8. Close
9. Apply POP (or External fixator but rarer)
10. Dress
11. Move off theatre table

**Tibial nailing (times will be on the SIGN nail form)**

1. Starting time
2. Re-debridement (step 6 & 7 above)
3. Approach
4. Fracture reduction
5. Reaming
6. Nail insertion
7. Nail locking
8. Close wounds
9. Dress
10. Move off theatre table

**Ward: dressing change**

1. Remove previous dressing
2. Clean wound
3. Apply new dressing

**Ward: medication dispensing**

1. Check patient details
2. Dispense medication
3. **Costs/required needed**

- Instrument costs for debridement (minor theatre and major theatre)
- Costs of Normal saline, gauze
- SIGN nail kit for internal fixation nail (major theatre)
- C-arm for major theatre (not applicable, discussed with JB, no Xrays for open tibias)
- Grade of staff (combined with QECH salaries in appendix)
- Medication costs
- Lab costs (Hb)
- Water and electricity
- SIGN nail timing sheets

1. **Interviews required**

AE:

- Triage nurse
- ATC doctor

Orthopaedic ward:

- Matron
- Nurse

Theatre:

- Scrub nurse
- Surgeon (registrar). Will need to ask the timing for debridement and flaps.
- Anaesthetist (OCO or registrar)

1. **Time motion analysis planned**

Ward:

- Dressing change by ward nurse
- Medication administration by ward nurse
- Post-operative physiotherapy exercises

Radiology:

- AP and lateral Xrays of post-op Xrays

| Description of facility: | | | | | | | | | | | | | | | | | |
| --- | --- | --- | --- | --- | --- | --- | --- | --- | --- | --- | --- | --- | --- | --- | --- | --- | --- |
|  | | | | | | | | | | | | | | | | | |
|  | | Mon | | Tues | | | Wed | | | Thurs | | Fri | | Sat | | | Sun |
| Operating hours | |  | |  | | |  | | |  | |  | |  | | |  |
| **Facility size (number of rooms available). A separate sheet is included to draw a map of the emergency rooms, orthopaedic ward, theatres and clinics in the facility** | | | | | | | | | | | | | | | | | |
| Waiting rooms | Clinic rooms | | | | | Offices | | |  | | | |  | |  | | |
|  |  | | | | |  | | |  | | | |  | |  | | |
| **What are the types of staff currently working in the facility?** | | | | | | | | | | | | | | | | | |
| Position | | | Unit | | Position | | | Unit | | | Position | | | | | Unit | |
| Doctors (D) | | |  | | Nurse (N) | | |  | | | Orthopaedic clinical officers (OCO) | | | | |  | |
| Volunteers (V) | | |  | | Physiotherapist (P) | | |  | | | Cleaners (C) | | | | |  | |
| Scrub nurse (SN) | | |  | | Radiographer (R) | | |  | | |  | | | | |  | |
| **How many staff does the clinic have?** | | | | | | | | | | | | | | | | | |
| Permanent | | |  | | Partner funded | | |  | | |  | | | | |  | |
| Locum/On Short Contracts | | |  | | volunteers | | |  | | |  | | | | |  | |

**Map of facilities**: This is a sketch of the physical layout of the facility

**Client Flow- Based on Observation/Interviews**

**Please describe a typical client visit. i.e. Who does the client see upon arrival at the facility and then what happens. E.g. Someone comes with an open tibia fracture. Attach room numbers etc.**

**Interview with Facility Manager**

**Brief introduction of service provider**

• **Name, title and duties at the clinic & How long has the interviewee been in the facility?**

**Client Flow**

**Description of a typical client visit from the Service Provider’s Point of View**

**Equipment in the facility’s rooms**

| **Room** | **Equipment** | **Quantity** |
| --- | --- | --- |
|  |  |  |
|  |  |  |
|  |  |  |
|  |  |  |
|  |  |  |
|  |  |  |
|  |  |  |
|  |  |  |
|  |  |  |
|  |  |  |
|  |  |  |
|  |  |  |
|  |  |  |
|  |  |  |
|  |  |  |
|  |  |  |
|  |  |  |
|  |  |  |
|  |  |  |
|  |  |  |

**Facility Personnel**

| **Initials of staff** | **Position or job title** | **Number of full working days in facility** |
| --- | --- | --- |
|  |  |  |
|  |  |  |
|  |  |  |
|  |  |  |
|  |  |  |
|  |  |  |
|  |  |  |
|  |  |  |
|  |  |  |
|  |  |  |
|  |  |  |
|  |  |  |
|  |  |  |
|  |  |  |
|  |  |  |

**Supplies used for open tibia fractures**

| Period | Units per month |
| --- | --- |
| Gloves |  |
| Aprons |  |
| Cleaning supplies |  |
| Registers |  |
| Cotton |  |
| Sharp boxes |  |
| Methylated spirits |  |
| other |  |
|  |  |
|  |  |
|  |  |
|  |  |
|  |  |
|  |  |

**Utilities**

| **Period** | **Water** | **Electricity** | **Fuel** | **Telephone** | **Insurance** | **Other** |
| --- | --- | --- | --- | --- | --- | --- |
| **Jan** |  |  |  |  |  |  |
| **Feb** |  |  |  |  |  |  |
| **Mar** |  |  |  |  |  |  |
| **Apr** |  |  |  |  |  |  |
| **May** |  |  |  |  |  |  |
| **Jun** |  |  |  |  |  |  |
| **Jul** |  |  |  |  |  |  |
| **Aug** |  |  |  |  |  |  |
| **Sept** |  |  |  |  |  |  |
| **Oct** |  |  |  |  |  |  |
| **Nov** |  |  |  |  |  |  |
| **Dec** |  |  |  |  |  |  |

**Waste Management**

| **Type of waste** | **Frequency of disposal** | **Waste collection company** | **Disposal Place** |
| --- | --- | --- | --- |
|  |  |  |  |
|  |  |  |  |
|  |  |  |  |
|  |  |  |  |
|  |  |  |  |
|  |  |  |  |
|  |  |  |  |
|  |  |  |  |
|  |  |  |  |

**Recurrent Training**

| **Personnel involved in training** | **Funded by** | **Fees** | **Travel** | **Subsistence + misc** |
| --- | --- | --- | --- | --- |
|  |  |  |  |  |
|  |  |  |  |  |
|  |  |  |  |  |
|  |  |  |  |  |
|  |  |  |  |  |
|  |  |  |  |  |
|  |  |  |  |  |
|  |  |  |  |  |
|  |  |  |  |  |

**Time and Motion**

| **Observation** | | | |
| --- | --- | --- | --- |
| **Start time** | | **End time** | |
| **HH** | **MM** | **HH** | **MM** |

**Grade of provider being observed:**

| **Staff grade** | **Check** |
| --- | --- |
| **Nurse** |  |
| **Scrub nurse** |  |
| **OCO** |  |

**Activity codes**

| **CODE** | **Activity description** |
| --- | --- |
| ADMIN | Booking patients into hospital system |
| XRAY | Time to get radiographs |
| ABX | Time to get antibiotics |
| TET | Time to tetanus |
| PMED | Time to get pain medication |
| IMOB | Time to apply immobilisation |
| SURG | Time to initial surgery |
| PXRAYS | Time to post op xrays |
| DISCH | Time to discharge |
|  |  |

| **Observation (procedure)** | **Staff** | **Start time** | **End Time** | **Resources** |
| --- | --- | --- | --- | --- |
| **1** |  |  |  |  |
| **2** |  |  |  |  |
| **3** |  |  |  |  |
| **4** |  |  |  |  |
| **5** |  |  |  |  |
| **6** |  |  |  |  |
| **7** |  |  |  |  |
| **8** |  |  |  |  |
| **9** |  |  |  |  |
| **10** |  |  |  |  |
| **11** |  |  |  |  |
| **12** |  |  |  |  |
| **13** |  |  |  |  |
| **14** |  |  |  |  |
| **15** |  |  |  |  |
| **16** |  |  |  |  |
| **17** |  |  |  |  |
| **18** |  |  |  |  |
| **19** |  |  |  |  |
| **20** |  |  |  |  |
| **21** |  |  |  |  |
| **22** |  |  |  |  |
| **23** |  |  |  |  |
| **24** |  |  |  |  |
| **25** |  |  |  |  |
| **26** |  |  |  |  |
| **27** |  |  |  |  |
| **28** |  |  |  |  |
| **29** |  |  |  |  |
| **30** |  |  |  |  |
| **31** |  |  |  |  |
| **32** |  |  |  |  |
| **33** |  |  |  |  |
| **34** |  |  |  |  |
| **35** |  |  |  |  |
| **36** |  |  |  |  |
| **37** |  |  |  |  |
| **38** |  |  |  |  |
| **39** |  |  |  |  |
| **40** |  |  |  |  |
| **41** |  |  |  |  |
| **42** |  |  |  |  |
| **43** |  |  |  |  |
| **44** |  |  |  |  |
| **45** |  |  |  |  |
| **46** |  |  |  |  |
| **47** |  |  |  |  |
| **48** |  |  |  |  |
| **49** |  |  |  |  |

**General observations**

**List of People (Positions and Contact details) talked to.**

| **Contact name** | **Position** | **Contact details** |
| --- | --- | --- |
|  |  |  |
|  |  |  |
|  |  |  |
|  |  |  |
|  |  |  |
|  |  |  |
|  |  |  |
|  |  |  |
|  |  |  |
