# Supplementary material for: Economic burden and cost-effectiveness of treatments for open tibia fractures in Malawi: Economic analysis of a multicentre prospective cohort study
Source: PLoS One. 2025 Sep 5;20(9):e0331569. doi: 10.1371/journal.pone.0331569 (PMC12413004; doi:10.1371/journal.pone.0331569)
Supplement: S2 File — (DOCX) [file pone.0331569.s002.docx]

Inclusivity in global research

PLOS’ policy on inclusivity in global research aims to improve transparency in the reporting of research performed outside of researchers’ own country or community and ensures that PLOS publications reporting global research adhere to high standards for research ethics and authorship. Authors of relevant research articles may be asked to complete the questionnaire below, which outlines ethical, cultural, and scientific considerations specific to inclusivity in global research. This questionnaire may be requested when researchers have travelled to a different country to conduct research, if research uses samples collected in another country, research with Indigenous populations or their lands, or if research is on cultural artefacts. Researchers travelling to another country solely to use laboratory equipment will not normally be required to complete the questionnaire. However, the questionnaire can be requested at the journal’s discretion for any submission – if you have been requested to complete this questionnaire by the PLOS journal you submitted to, please do so.

Please complete the questionnaire below and include this as a Supporting Information file with your manuscript. Note that if your paper is accepted for publication, this checklist will be published with your article in the supporting information files. Please ensure that you reference the checklist in the main body of your manuscript. We suggest adding a subsection ‘Inclusivity in global research’ to your Methods section and adding the following sentence: “Additional information regarding the ethical, cultural, and scientific considerations specific to inclusivity in global research is included in the Supporting Information (SX Checklist)”

The questions have been designed to be applicable to a wide range of study types, and there are subsections for both human subjects research and non-human subjects research. If any of the questions are not relevant to your research please mark them as “N/A” as appropriate.

**Ethical considerations, permits and authorship**

*This section is applicable to all research types.*

Provide details as to who granted permissions and/or consent for the study to take place in the Methods section of your manuscript. This should include the names of **all** ethics boards, governmental organizations, community leaders or other bodies that provided approval for the study. If individuals provided approval refer to these people by their role or title but do not list their name(s).

“The study was approved by the College of Medicine Research and Ethics Committee (COMREC Ref number: P.09/20/3130) in Malawi, and the Liverpool School of Tropical Medicine Research Ethics Committee (Reference number: 20-068). Written informed consent was obtained from all participants. “

Reported on page number: 10

If there were any deviations from the study protocol after approval was obtained please provide details of these changes in the Methods section of your manuscript.
Did this study involve local collaborators that are residents of the country where the research was conducted or members of the community studied? If you do not have any authors from said communities, please provide an explanation for this below.

MS is an orthopaedic clinical officer from Malawi.

NN and MC are Malawian orthopaedic surgeons, while NB, originally from the Democratic Republic of Congo, has been practising orthopaedic surgery in Malawi for over 25 years.

All four—MS, NN, MC, and NB—played a key role in shaping the study. They facilitated a series of stakeholder meetings across Malawi, engaging a wide range of local healthcare professionals including orthopaedic surgeons, anaesthetists, clinical officers, nurses, and patients.

No deviation

Reported on page number: NA

Everyone listed as an author should meet PLOS’ criteria for authorship and all individuals who meet these criteria should be included in the author byline, rather than the acknowledgements. For further information please see the journal’s Authorship Policy.

**Human subjects research (e.g. health research, medical research, cross-cultural psychology)**

Did you obtain written informed consent from a representative of the local community or region before the research took place? How did you establish who speaks for the community? Details of written informed consent obtained from study participants should be reported separately in the Methods section of your manuscript.

Written informed consent was obtained from all participants. The questionnaire interviews were conducted by research assistants who had no formal ties to any of the six hospitals involved in the study.

How did members of the local community provide input on the aims of the research investigation, its methodology, and its anticipated outcome(s)?

Interim findings were shared with the orthopaedic department roughly every four months to encourage discussion and gather input. MB, NN, CM, and LB reviewed these early results and contributed recommendations on which variables should be explored further and included in the final analysis. Their orthopaedic expertise was instrumental in identifying key aspects worth examining. This joint process of reviewing and interpreting the data helped ensure a thorough and thoughtful analysis. The involvement of regionally based researchers added depth to the interpretation, ensuring that the analysis reflected contextually relevant insights and priorities.

When engaging with the local community, how did you ensure that the informed consent documents and other materials could be understood by local stakeholders?

To ensure that informed consent documents and related materials were accessible to local stakeholders, patient information leaflets were translated into the local language. For participants who were illiterate, the content was read aloud to them by a member of the research team to ensure full understanding before consent was obtained.

During the study design phase, the team worked closely with the Malawi-Liverpool-Wellcome Trust (MLW) Media and Communication department. This collaboration provided essential guidance on how to address cultural and religious sensitivities within the study population. Their input helped the team tailor materials and approaches to ensure they were appropriate, respectful, and aligned with local traditions and beliefs.

Additionally, the MLW ethics department was involved in overseeing the ethical aspects of the study. Their role included advising on how to approach sensitive issues and ensuring that all materials and processes upheld ethical standards, particularly when engaging with diverse communities.

Will the findings of the research be made available in an understandable format to stakeholders in the community where the study was conducted (e.g. via a presentation, summary report, copies of publications, etc.)? Please provide details of how this will be achieved.

To raise public awareness and share key findings, the research team partnered with various media platforms—including radio, TV, and newspapers—to organise a Road Traffic Awareness Week. This initiative helped bring the study’s insights to the wider public, using accessible and far-reaching communication channels. By working with established media outlets, the team was able to extend the impact of the research beyond the academic community and into everyday conversations.

Links to media coverage:

- [Malawi Orthopaedic Association Website](https://malawiorthopaedic.mw/)
- [Times360 Malawi Twitter](https://twitter.com/Times360Malawi/status/1437360018584637440)
- [YouTube – Malawi Orthopaedic Association](https://www.youtube.com/watch?v=yjFbXkR7m2s&ab_channel=MalawiOrthopaedicAssociation)

In addition to public outreach, the research team created brief presentations and infographics summarising the findings, which were shared with clinical officers at district hospitals. These were displayed in hospital settings and presented during morning meetings, ensuring frontline health workers could easily access and engage with the information in their day-to-day work.

The study also prioritised sharing results with all orthopaedic professionals involved in the project. This included dissemination through stakeholder meetings, educational workshops, and postgraduate sessions. Engaging directly with clinicians helped support the translation of research into practice, reinforcing the relevance of the findings and their application in improving patient outcomes.

**Non-human subjects research using specimens/ animals collected as part of the study, or those housed in archival collections. Examples include archaeology, paleontology, botany and zoology.**

Did the permission you obtained from a local authority to perform the study include an agreement on access to outputs and benefit sharing? This may include procedures to enable fair distribution of the benefits and resources arising from the research performed. Please include any details of Prior Informed Consent and Benefit Sharing Agreements obtained. These may be required by field-specific regulations, for example the Convention on Biological Diversity (CBD) and the associated Nagoya Protocol.

Not applicable

If the material used in your study was imported, please A) provide the year it was imported and B) indicate whether permits were obtained to import/export the materials used, C) provide details of any permits obtained. If this information is not available, please indicate this.

Not applicable, this is a prospective cohort study.

If you used archival specimens, please state how the material used in your study was acquired by the institute it is held in and provide details of any permits obtained for the original excavations/ sample collection. If this information is not available, please indicate this.

Not applicable, this is a prospective cohort study.

How was the potential cultural significance of the materials collected in your study to local communities considered in your research design? Were Indigenous peoples and/or local researchers and institutions involved with archaeological excavations / collection of specimens? If so, please provide a description of their involvement.

Not applicable, this is a prospective cohort study.

If your manuscript includes photographs of human remains please indicate whether authors obtained permission from descendants or affiliated cultural communities to do so.

Not applicable, this is a prospective cohort study.
