# Supplementary material for: Economic burden and cost-effectiveness of treatments for open tibia fractures in Malawi: Economic analysis of a multicentre prospective cohort study
Source: PLoS One. 2025 Sep 5;20(9):e0331569. doi: 10.1371/journal.pone.0331569 (PMC12413004; doi:10.1371/journal.pone.0331569)
Supplement: Attachment — Submitted filename: PLOS_one_revisions_4.4.25.docx [file pone.0331569.s003.docx]

Wednesday, 4^th^ April 2025

Dear Professor Chenette,

**Re: PONE-D-24-40054 revision**

I write on behalf of my co-authors to thank the reviewers for their careful review and appreciate the opportunity to respond with a revised submission.

The authors have declared that no competing interests exist.

Please see the point-by-point responses below.

Yours Sincerely,

Alexander Thomas Schade

(On behalf of the authors)

1. **Reviewer 1**

**Comment #1:**

The study suggests that intramedullary nailing is the most cost-effective treatment for open tibia fractures in Malawi. However, the practical implementation of this finding might require further discussion:

- Training of orthopedic surgeons: Given the shortage of trained orthopedic surgeons in Malawi, the feasibility of expanding surgical treatments should be addressed. If intramedullary nailing is to be widely adopted, investment in surgeon training and infrastructure is necessary.

- Accessibility of surgical care: Since most fractures in Malawi are treated non-operatively by Clinical Officers, the study should discuss whether a shift toward surgical management is realistically achievable within the current healthcare system.

These aspects would strengthen the clinical relevance of the study and help translate findings into practical recommendations.

**Author’s Response:** Thank you for this very important comment and we have developed this further in our manuscript discussion. We were not able to include the costs of training or infrastructure in this manuscript and have acknowledged this limitation. We have further emphasised that others have shown that surgical training needs to be increased to match the burden of injury, but it is unclear how feasible this is. This study adds to the growing evidence that the societal costs of treating injuries non-operatively are too great to ignore.

Lines: 337

“We did not consider the costs of building theatres or surgical training, which might increase the societal costs further. In Malawi, improved surgical training and expanding surgical capacity to manage the injury burden has been shown to reduce amputation rates (1). The Lancet Commission on Global Surgery reported a global shortage of 1.1 million surgical and anaesthetic providers in 2015, most of whom are required in low- and middle-income countries (LMICs)(2). The Commission's recommendation was to expand the surgical and anaesthetic workforce to at least a minimum density of 20 providers for every 100,000 population by 2030, as lower densities were correlated with increased maternal mortality (3). Meeting this goal would involve enrolling an extra 1.27 million providers and a total investment of about $45 billion—an aspiration that is perhaps unattainable for most LMICs considering current resource limitations.

Despite this, the high societal and economic price for treating injuries conservatively is too great. The WHO Economic Cost of Ill-Health Model estimates that an economic loss from unattended surgical conditions of $20.7 trillion will occur in 128 countries from 2015 to 2030, with over half ($12.3 trillion) occurring in LMICs (4).”

**Journal requirements**

**Comment #1:**

 When submitting your revision, we need you to address these additional requirements.

Please ensure that your manuscript meets PLOS ONE's style requirements, including those for file naming.

**Author’s Response:**

Thank you, we have changed the style requirements

**Comment #2:**

Please include a complete copy of PLOS’ questionnaire on inclusivity in global research in your revised manuscript.

**Author’s Response:**

Many thanks, we have added this to the revision as a supplement.

**Comment #3:**

We note that the grant information you provided in the ‘Funding Information’ and ‘Financial Disclosure’ sections do not match. When you resubmit, please ensure that you provide the correct grant numbers for the awards you received for your study in the ‘Funding Information’ section.

**Author’s Response:**

Many thanks, we have corrected this so that the Funding information and financial disclosures match.

Line 205

“This research was funded in whole by Wellcome [Grant number 203919]. For the purpose of open access, the author has applied a CC BY public copyright licence to any Author Accepted Manuscript version arising from this submission.”

**Comment #4:**

Thank you for stating the following in your Competing Interests section: “no. competing interests.”
Please complete your Competing Interests on the online submission form to state any Competing Interests. If you have no competing interests, please state "The authors have declared that no competing interests exist.", as detailed online in our guide for authors at <http://journals.plos.org/plosone/s/submit-now> This information should be included in your cover letter; we will change the online submission form on your behalf.

**Author’s Response:** Thank you for this comment, we have added that “The authors have declared that no competing interests exist” in our cover letter

**Comment #5**

Thank you for uploading your study's underlying data set. Unfortunately, the repository you have noted in your Data Availability statement does not qualify as an acceptable data repository according to PLOS's standards. At this time, please upload the minimal data set necessary to replicate your study's findings to a stable, public repository (such as figshare or Dryad) and provide us with the relevant URLs, DOIs, or accession numbers that may be used to access these data. For a list of recommended repositories and additional information on PLOS standards for data deposition, please see [https://journals.plos.org/plosone/s/recommended-repositories](https://track.editorialmanager.com/CL0/https:%2F%2Fjournals.plos.org%2Fplosone%2Fs%2Frecommended-repositories/1/010f01960032d63b-e5f006f3-2c10-44b1-b748-6d7073d2f679-000000/oulYsj4ONcZITniVc48QirFhy7SOWlAtQklv3Pye51s=205).

**Author’s Response:**

Thanks, we have uploaded the dataset to Open Science Framework which is one of the Cross-disciplinary repositories

**Comment #6**

Please note that in order to use the direct billing option the corresponding author must be affiliated with the chosen institute. Please either amend your manuscript to change the affiliation or corresponding author, or email us at [plosone@plos.org](mailto:plosone@plos.org) with a request to remove this option.

**Author’s Response:**

Thanks, the corresponding author is affiliated with The Liverpool School of Tropical medicine.

**Comment #7**

Please review your reference list to ensure that it is complete and correct. If you have cited papers that have been retracted, please include the rationale for doing so in the manuscript text, or remove these references and replace them with relevant current references. Any changes to the reference list should be mentioned in the rebuttal letter that accompanies your revised manuscript. If you need to cite a retracted article, indicate the article’s retracted status in the References list and also include a citation and full reference for the retraction notice.

**Author’s Response:**

Thanks, this has been corrected.

1.	Young S, Banza L, Mkandawire N. The impact of long term institutional collaboration in surgical training on trauma care in Malawi. Springerplus. 2016;5:407.

2.	Meara JG, Leather AJM, Hagander L, Alkire BC, Alonso N, Ameh EA, et al. Global Surgery 2030: evidence and solutions for achieving health, welfare, and economic development. The Lancet. 2015;386(9993):569-624.

3.	Lozano R, Naghavi M, Foreman K, Lim S, Shibuya K, Aboyans V, et al. Global and regional mortality from 235 causes of death for 20 age groups in 1990 and 2010: a systematic analysis for the Global Burden of Disease Study 2010. The Lancet. 2012;380(9859):2095-128.

4.	Bloom D, Cafiero-Fonseca E, Jane Llopis E, Abrahams-Gessel S, Bloom L, Fathima S, et al. The Global Economic Burden of Noncommunicable Diseases2011.
