# Supplementary material for: Economic burden and cost-effectiveness of treatments for open tibia fractures in Malawi: Economic analysis of a multicentre prospective cohort study
Source: PLoS One. 2025 Sep 5;20(9):e0331569. doi: 10.1371/journal.pone.0331569 (PMC12413004; doi:10.1371/journal.pone.0331569)
Supplement: Attachment — Submitted filename: review for PlosOne.docx [file pone.0331569.s004.docx]

Abstract

- Open tibia fracture can have an equal impact on women, what makes it more detrimental to men according to your research?
- We obtained quality adjusted life years (QALYs) from the EuroQoL 5 Dimension 3 Level (EQ-5D-3L)…
- Why were costs reported in 2021; at least inflate to 2024, or better still, report using 2024 costs.

Background

Method

- The study protocol was published -------include date it was published. Ln 99
- Write the statement to make meaning or remove the bracket and let it be a complete sentence: At recruitment …. study. Lns 133-135
- Each procedure was observed between 1-3 times. - What was observed? Ln 154
- Did the difference in the way the costs were collected impact the study?
- How was missing data inputted?

Result

- What was the incremental change in Costs and QALYs. Can the QALY per treatment type and location (central vs district) be included? What is the difference between costs and QALYs based on where the treatment took place?
- Can you include a table showing the costs, QALYs, Incremental Costs, Incremental QALYs and ICER values
