# Supplementary material for: Economic burden and cost-effectiveness of treatments for open tibia fractures in Malawi: Economic analysis of a multicentre prospective cohort study
Source: PLoS One. 2025 Sep 5;20(9):e0331569. doi: 10.1371/journal.pone.0331569 (PMC12413004; doi:10.1371/journal.pone.0331569)
Supplement: Attachment — Submitted filename: PLOSONE_responses.docx [file pone.0331569.s005.docx]

Wednesday, 4^th^ April 2025

Dear Professor Chenette,

**Re: PONE-D-24-40054R1**

I write on behalf of my co-authors to thank the reviewers for their careful review and appreciate the opportunity to respond with a revised submission.

The authors have declared that no competing interests exist.

This research was funded in whole by Wellcome [Grant number 203919]. For the purpose of open access, the author has applied a CC BY public copyright licence to any Author Accepted Manuscript version arising from this submission. The funders had no role in study design, data collection and analysis, decision to publish, or preparation of the manuscript

Please see the point-by-point responses below.

Yours Sincerely,

Alexander Thomas Schade

(On behalf of the authors)

1. **Reviewer 2**

**Comment #1:**

**Reviewer #2: While I give kudos to the authors, I think the discussion could be more robust judging by data analysis provided. So, I suggest the author beef up the discussion section with relevant citations. This will justify the data analysis provided.**

**Author’s Response:** We thank the reviewer for their positive feedback and constructive suggestion. In response, we have substantially revised and expanded the Discussion section to better contextualise our findings and strengthen the link between the data analysis and its implications. Specifically, we have incorporated additional references to relevant literature (1-4), including recent studies in similar settings, to support our interpretations and highlight the significance of our results.

Lines: 275

“Orthopaedic interventions might not be affordable to the Malawi government, but substantial investment in trauma care will be required from government and stakeholders to reduce the large societal impact of injury (1)”

Line 296

Open tibia fractures have devastating economic consequences on patients and their households too (2).

Line 313

Further research should focus on factors and outcomes that improve the return of income such as social support and rehabilitation on injury (3).

Line 315

As LMIC economies grow and motor transport becomes more common, fractures are placing a large financial strain on the economies (4).

”

**Reviewer #3:**

**Comment #1:**

**Open tibia fracture can have an equal impact on women, what makes it more detrimental to men according to your research?**

**Author’s Response:** Thank you for your comment. Open tibia fractures can indeed have a significant impact on both men and women. However, in our cohort, 84% of patients were male, which is consistent with global data showing that traumatic injuries disproportionately affect young men (4). In the Malawian context, cultural norms often position men as the primary income earners (5). As a result, the economic consequences of injury may be more pronounced for men, particularly in terms of lost income and reduced household stability. To our knowledge, this socioeconomic impact has not been previously documented in this setting and warrants further investigation.

**Comment #2:**

**We obtained quality adjusted life years (QALYs) from the EuroQoL 5 Dimension 3 Level (EQ-5D-3L)…**

**Author’s Response:** Many thanks, we have amended the abstract accordingly.

**Comment #2:**

**Why were costs reported in 2021; at least inflate to 2024, or better still, report using 2024 costs.**

**Author’s Response:** Thank you for your comment. As outlined in the methods section, recruitment took place between 12 February 2021 and 15 March 2022. To reflect the timing of data collection and ensure consistency, costs were reported in 2021 values.

**Comment #3:**

**The study protocol was published -------include date it was published. Ln 99**

**Author’s Response:** Thank you for highlighting this. The study protocol was published in 2021, and we have now included the publication year at line 99 in the Methods section for clarity.

Lines: 100

“The study protocol was previously published in 2021”

**Comment #3:**

**Write the statement to make meaning or remove the bracket and let it be a complete sentence: At recruitment …. study. Lns 133-135**

**Author’s Response:** Thank you for your suggestion. We have revised and completed the sentence for clarity.

Line 134

“At recruitment, participants were asked to retrospectively complete the questionnaire for their health state pre-injury and at each subsequent follow-up visit, study.”

**Comment #3:**

**Each procedure was observed between 1-3 times. - What was observed? Ln 154**

**Author’s Response:** Thank you for highlighting this point. We have revised the manuscript to clarify what aspects of the procedure were observed. Specifically, we have added a description of the patient treatment pathway in the main text and detailed which procedures were directly observed in Appendix A. This should make the scope and focus of the observations clearer to the reader.

**Comment #3:**

**- Did the difference in the way the costs were collected impact the study?**

**Author’s Response:** Thank you for your comment. Due to the lower frequency of open tibia fractures in district hospitals, we were unable to conduct time-and-motion analyses at those sites. This difference in data collection methods may have introduced some limitations, which we have now acknowledged and discussed in the limitations section of the manuscript.

**Comment #4
- How was missing data inputted?**

Thank you for your question. Observations with missing data were excluded from the relevant analyses. Given the small number of missing values, we did not attempt formal imputation. Where appropriate, we conducted sensitivity analyses to assess the potential impact of missing data on our findings.

**Comment #5**

**- What was the incremental change in Costs and QALYs. Can the QALY per treatment type and location (central vs district) be included? What is the difference between costs and QALYs based on where the treatment took place?**

**- Can you include a table showing the costs, QALYs, Incremental Costs, Incremental QALYs and ICER values**

Thank you for your helpful comments. As clarified in the revised manuscript, Table 2 presents the total costs, incremental costs, and cost per QALY (ICER) for each treatment type, stratified by treatment location (central vs district hospitals). This allows for direct comparison of cost-effectiveness across settings. QALYs and incremental QALYs were reported in our previous publication (7) and are not reproduced in full here to avoid duplication. However, the ICER values in Table 2 incorporate these published QALY estimates to provide a comprehensive assessment of cost-utility.

1.	McGuire F, Revill P, Twea P, Mohan S, Manthalu G, Smith PC. Recommendations for the development of a health sector resource allocation formula in Malawi. York: University of York; 2018.

2.	Schade AT, Sibande W, Kumwenda M, Desmond N, Chokotho L, Karasouli E, et al. "Don't rush into thinking of walking again": Patient views of treatment and disability following an open tibia fracture in Malawi. Wellcome Open Res. 2022;7:204.

3.	Cancelliere C, Donovan J, Stochkendahl MJ, Biscardi M, Ammendolia C, Myburgh C, et al. Factors affecting return to work after injury or illness: best evidence synthesis of systematic reviews. Chiropractic & Manual Therapies. 2016;24(1):32.

4.	World Health Organization. Global status report on road safety 2018. Geneva; 2018. Contract No.: CC BYNC-SA 3.0 IGO.

5.	National_Statistical_Office. Malawi Population and Housing Census report 2018. Zomba, Malawi: National_Statistical_Office; 2018.

6.	Bürkner P-C. Brms: An R package for Bayesian multilevel models using Stan. J Stat Softw. 2017;80(1).

7.	Schade AT, Sabawo M, Nyamulani N, Mpanga CC, Ngoie LB, Metcalfe AJ, et al. Functional outcomes and quality of life at 1-year follow-up after an open tibia fracture in Malawi: a multicentre, prospective cohort study. The Lancet Global Health. 2023.
